# Corticosteroid Is Associated with Both Hip Fracture and Fracture-Unrelated Arthropathy

**DOI:** 10.1371/journal.pone.0169468

**Published:** 2017-01-26

**Authors:** Feng-Chen Kao, Yao-Chun Hsu, Chien-Fu Jeff Lin, Ying-Ying Lo, Yuan-Kun Tu

**Affiliations:** 1 Department of Orthopaedics, E-Da Hospital, Kaohsiung, Taiwan; 2 School of Medicine for International Students, I-Shou University, Kaohsiung, Taiwan; 3 Department of Internal Medicine, E-Da Hospital, Kaohsiung, Taiwan; 4 Center for Database Research, E-Da Hospital, Kaohsiung, Taiwan; 5 Graduate Institute of Clinical Medicine, China Medical University, Taichung; Taiwan; 6 Department of Statistics, National Taipei University, Taipei, Taiwan; 7 Department of Orthopedic Surgery, Wan Fang Hospital, Taipei, Taiwan; 8 Department of Healthcare Administration, I-Shou University, Kaohsiung, Taiwan; Kaohsiung Medical University Hospital, TAIWAN

## Abstract

**Objective:**

We aimed to investigate whether and how corticosteroid use was associated with serious hip arthropathy.

**Methods and Materials:**

This population-based cohort study analyzed the Taiwan National Health Insurance Research Database and screened the one-million random sample from the entire population for eligibility. The steroid cohort consisted of 21,995 individuals who had used systemic corticosteroid for a minimum of 6 months between January 1, 1997 and December 31, 2006. They were matched 1:1 in propensity score on the index calendar date with controls who never used steroid. All participants were followed up until occurrence of serious hip arthropathy that required arthroplasty, withdrawal from the national health insurance, or the end of 2011. Surgical indication was classified as fracture-related and -unrelated. The cumulative incidence of hip arthroplasty was estimated by the Kaplan Meier method. The association with steroid exposure was explored by the Cox proportional hazard model.

**Results:**

Cumulative incidences of hip arthroplasty after 12 years of follow-up were 2.96% (95% confidence interval [CI], 2.73–3.2%) and 1.34% (95% CI, 1.2–1.51%) in the steroid users and non-users, respectively (*P*<0.0001). The difference was evident in fracture-related arthroplasty with 1.89% (95% CI, 1.71–2.09%) versus 1.10% (95% CI, 0.97–1.25%), but more pronounced in fracture-unrelated surgery, 1.09% (95% CI, 0.95–1.24%) versus 0.24% (95% CI, 0.19–0.32%). Multivariate-adjusted Cox regression analysis confirmed steroid use was independently associated with both fracture-related (adjusted hazard ratio [HR], 1.65; 95% CI, 1.43–1.91) and unrelated arthroplasty (adjusted HR, 4.21; 95% CI, 3.2–5.53). Moreover, the risk for fracture-unrelated arthropathy rose with steroid dosage, as the adjusted HR increased from 3.30 (95% CI, 2.44–4.46) in the low-dose subgroup, 4.54 (95% CI, 3.05–6.77) in intermediate-dose users, to 6.54 (95% CI, 4.74–9.02) in the high-dose counterpart (*P*_*trend*_<0.0001).

**Conclusions:**

Corticosteroid use is associated with long-term risk of hip arthroplasty, particularly for fracture-unrelated arthropathy.

## Introduction

**C**orticosteroid may induce bone disease that is associated with substantial morbidity and mortality[1]. For instance, osteoporosis is a well-recognized complication that can precipitate fracture of the weight-bearing bones [2, 3]. Previous studies have established that glucocorticoid use is a risk factor of vertebral and hip fracture[4]. However, the detrimental effect of corticosteroid on bone health extends beyond demineralization. It may also reduce osteocytes’ life span, impair the balance between osteoclast and osteoblast, and compromise skeletal angiogenesis[5–7]. As another complication of steroid exposure, osteonecrosis reportedly affects femoral heads in 8~10% of the users[8]. An epidemiological survey from Japan estimated that among 2,500~3,300 new cases of non-traumatic osteonecrosis every year, 34.7% were induced by corticosteroid[9]. How steroid duration, dosing frequency, and cumulated dosage might impact the risk of osteonecrosis, however, remains controversial[10–12].

Management for hip osteonecrosis depends on severity of the disease. A replacement arthroplasty is warranted for a symptomatic joint that is extensively degenerated or destructed[13]. The surgery of choice reflects the principle of “different operations for different stages” [14]. A hemi-arthroplasty is often sufficient for an intact acetabulum, but a total hip arthroplasty (THA) is usually required for a substantially degenerated joint[15]. It has been estimated that 5~12% of all THAs result from osteonecrosis[16], but how it relates to corticosteroid use has not been elucidated. Prior researches were predominantly hospital-based, without population-scale data. Besides, previous studies mostly focused on fracture-related outcomes and paid less attention to arthropathy unrelated to bone fracture.

In order to clarify the association between corticosteroid and risk of hip arthropathy, this population-based study analyzed a national healthcare research database which covers nearly the entire population of Taiwan. Individuals with and those without exposure to corticosteroid were followed up for the occurrence of hip arthroplasty over a 12-year study period.

## Methods

### Study design and data source

This is a retrospective open-cohort study based on analysis of the National Health Insurance Research Database (NHIRD). In Taiwan, the Ministry of Health and Welfare had launched the National Health Insurance (NHI) program since 1995 to cover healthcare of the 22.9 million residents in this country. Because the NHI is a single-payer system that implements compulsory and universal policy, it reimburses more than 99% of the national population. The Taiwan National Health Research Institute (NHRI) establishes and maintains NHIRD, which contains comprehensive registry and claim data for research purpose. In the NHIRD, diseases were coded according to the International Classification of Diseases, 9^th^ Revision, Clinical Modification [ICD-9-CM]. Our previous studies have demonstrated utility of the NHIRD in population-based research[17, 18]. In addition to the original NHIRD, the NHRI constructed and maintained the Longitudinal Health Insurance Database (LHID) as a representative data subset of all NHI enrollees. The expanded LHID 2000 was composed of 1,000,000 individuals randomly sampled from the years 1997 through 2011 registry of all beneficiaries. Its representativeness is supported by similar distribution in age, gender, and healthcare cost with the original NHIRD. Data acquisition and study conduction were approved by the institutional review board of E-Da Hospital (EMRP-103-011; EMRP-103-012) as well as Taiwan NHRI (NHIRD-103-116). The study was exempted from a full review by the institutional review board of the E-Da Hospital.

### Definition of the study cohorts and outcomes

This cohort study analyzed the expanded LHID 2000 to identify study cohorts. Individuals exposed to oral or injected form of systemic corticosteroid for a minimum of 6 months between January 1, 1997 and December 31, 2006 were screened. The index day for baseline matching and observation inception was assigned at the date when corticosteroid had been used for 6 months. These steroid users were then matched 1:1 in the propensity score on the index day with controls who had not been exposed to any dose of corticosteroid. Individuals who had had hip hemiarthroplasty, total hip arthroplasty or hip joint disarticulation operated prior to or within 6 months after the index day were excluded from the study. Patients who were diagnosed with femoral neck fracture, femoral head fracture, acetabulum fracture, avascular necrosis (AVN) of femoral head, osteoarthritis (OA), rheumatoid arthritis (RA) or systemic lupus erythematous (SLE) before the index date were also excluded, given that these disorders might confound the association between steroid and hip arthroplasty. The corresponding disease and procedure codes were listed in the Appendix (S1 Table).

All participants were followed up until the day when hip arthroplasty was performed, withdrawal from the NHI, or the end of 2011. Therefore, without occurrence of the study outcome or termination from the NHI enlistment, the follow-up duration was at least five years. The indication for arthroplasty was categorized by whether or not it was related to fracture. Fracture-related arthroplasty was defined when the admission diagnosis included a relevant code of bone fracture (femoral neck fracture, femoral head fracture, acetabulum fracture, and traumatic injury from falling down). Otherwise the indication was defined as fracture-unrelated and further classified as OA or AVN.

### Assessment of and adjustment for confounding factors

Comorbidity that could confound steroid use and hip arthropathy was considered. The analysis was adjusted for baseline conditions including cerebrovascular accident (CVA), osteoporosis[19], diabetes mellitus (DM) [20], cirrhosis[21], and chronic obstructive pulmonary disease (COPD). In order to delineate the dose-dependent relationship[12], the steroid users were grouped according to the dosage. All forms of systemic steroid reimbursed in the NHIRD were identified by the Anatomical Therapeutic Chemical codes (S2 Table, Appendix). The high-dose (upper quartile) subgroup indicated an average dose above 13.35mg/day, intermediate-dose (interquartile) between 1.09 and 13.35mg, and the low-dose (lower quartile) below 1.08mg per day.

### Statistical analysis

Continuous variables were summarized in mean and standard deviation, and categorical variables in number and proportion. The propensity score for steroid use was generated by the logistic regression built on baseline characteristics that could either affect steroid prescription or arthropathy risk, and included age, gender, osteoporosis, CVA, DM, liver cirrhosis, and COPD. The cumulative incidences of hip arthroplasty were estimated by the Kaplan-Meier method, and the between-cohort difference was tested by the 2-tailed log-rank test. The association between steroid exposure and fracture-unrelated hip arthroplasty was explored by the Cox proportional hazard model that took into account age, gender, and baseline comorbidity. Univariate and multivariate-adjusted hazard ratios (HRs) were reported. Dose-response relationship was evaluated by the Cochran–Armitage trend test. Data management and calculation of HRs were performed using the Statistical Package for the Social Sciences (version 17.0; SPSS Inc, Chicago, IL). Calculated results were expressed with the point estimates along with their 95% confidence intervals (CIs). All statistical tests were defined as significant with a *P* value less than 0.05.

## Results

### Baseline characteristics of the study population

From 1,000,000 individuals randomly sampled from the entire Taiwan population, we identified 21,995 patients free of any exclusion criteria (steroid cohort) and matched them in propensity score with 21,995 individuals who had not been exposed to systemic corticosteroid (non-steroid cohort). Baseline characteristics were comparable between the two study cohorts (Table 1).

**Table 1:** Baseline characteristics and primary outcomes of the study participants.

	Steroid users(N = 21,995)	Non users(N = 21,995)
Age, years (mean ± standard deviation)	45.89±23.06	45.96±23.06
Age group		
<20 years	3738(16.99)	3730(16.96)
20–39 years	3740(17.00)	3726(16.94)
40–59 years	7106(32.31)	7113(32.34)
60–79 years	7012(31.88)	7023(31.93)
> = 80 years	399(1.81)	403(1.83)
Gender		
Female, n (%)	10295(46.81)	10295(46.81)
Male, n (%)	11700(53.19)	11700(53.19)
Comorbidities		
Osteoporosis, n (%)	137(0.62)	132(0.6)
Cerebral vascular accident, n (%)	268(1.22)	268(1.22)
Diabetes mellitus, n (%)	403(1.83)	403(1.83)
Liver cirrhosis, n (%)	256(1.16)	261(1.19)
Chronic lung disease, n (%)	259(1.18)	259(1.18)
Hip arthroplasty, n (%)	711(3.23)	372(1.69)
Fracture, n (%)	456(1.99)	308(1.40)
Fracture-unrelated, n (%)	255(1.16)	64(0.29)
Osteoarthritis, n (%)	7(0.03)	12(0.05)
Avascular necrosis, n (%)	248(1.13)	52(0.24)
Follow-up year (mean, range)	13.11 (0.03–16.00)	13.69(0.16–16.00)

Note: Baseline characteristics did not significantly differ between the study cohorts

### Incidences of hip arthroplasty between study cohorts

**Fig 1:**
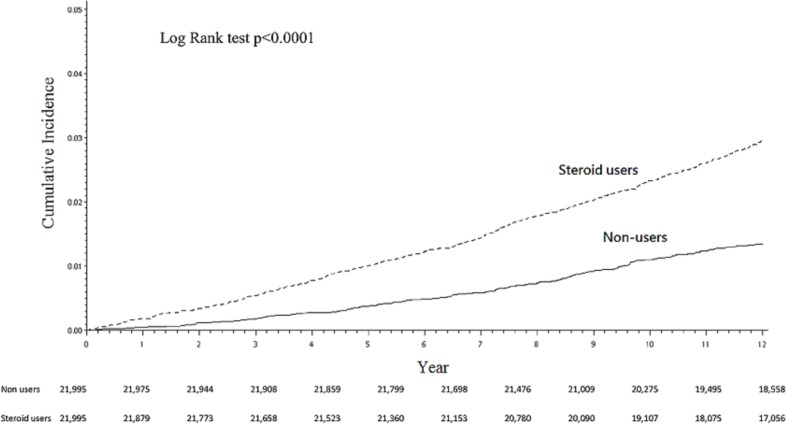
Twelve-year cumulative incidence of overall hip arthroplasty between the corticosteroid users and the non-users.

**Fig 2:**
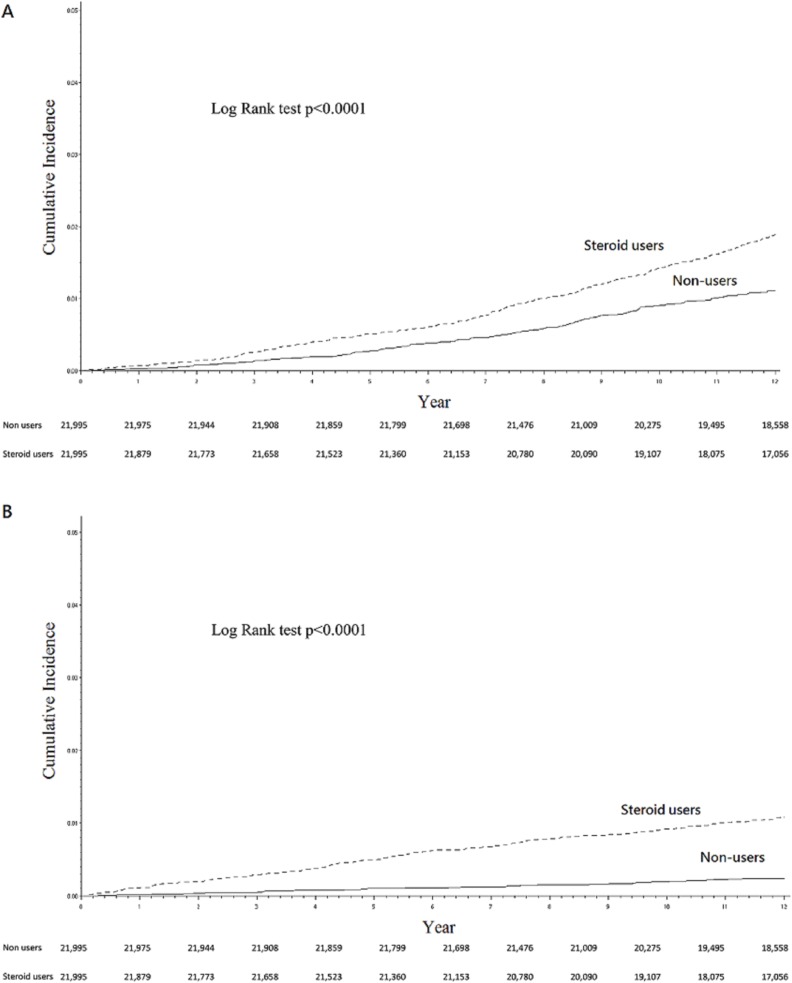
The steroid cohort had a higher incidence of hip arthroplasty not only for bony fracture (2A, upper panel) but also for fracture-unrelated arthropathy (2B, lower panel).

The steroid and non-steroid cohorts were followed up for a mean duration of 13.11 (range, 0.03–16.00) and 13.69 (range, 0.16–16.00) years, respectively. During the observation period, 711 (3.23%) corticosteroid users and 372 (1.40%) non-users underwent hip arthroplasty (Table 1). Cumulatively at 12 years, 2.96% (95% CI, 2.73–3.20%) in the steroid users received hip arthroplasty, significantly higher (*P*<0.0001) than 1.34% (95% CI, 1.20–1.51%) in the non-users (Fig 1). The difference between the steroid and non-steroid cohorts was evident in fracture-related arthroplasty (Fig 2A), 1.89% (95% CI, 1.71–2.09%) versus 1.10% (95% CI, 0.97–1.25%), but even more pronounced in fracture-unrelated surgery (Fig 2B), 1.09% (95% CI, 0.95–1.24%) versus 0.24% (95% CI, 0.19–0.32%).

### Multivariate-adjusted association between corticosteroid use and hip arthroplasty

In the unadjusted analysis, the risk for receiving hip arthroplasty was associated with steroid exposure, older age, osteoporosis, CVA, DM, liver cirrhosis, and COPD (Table 2). The multivariate-adjusted Cox proportional hazard model confirmed that steroid use (adjusted HR, 2.09; 95% CI, 1.84–2.37), age (adjusted HR, 1.05 per year; 95% CI, 1.05–1.06), osteoporosis (adjusted HR, 2.18; 95% CI, 1.37–3.45), and DM (HR, 1.52; 95% CI, 1.08–2.13) were independent risk factors for hip arthropathy that necessitated surgical treatment (Table 2).

**Table 2:** Univariate and multivariate analyses for hip arthropathy.

	Univariate		Multivariate	
	Crude HR	*P*	Adjusted HR	*P*
Corticosteroid use	2.01(1.77–2.28)	< .0001	2.09(1.84–2.37)	< .0001
Age (per year)	1.05(1.05–1.06)	< .0001	1.05(1.05–1.06)	< .0001
Osteoporosis	4.12(2.62–6.49)	< .0001	2.18(1.37–3.45)	0.0009
Cerebral vascular accident	1.75(1.05–2.92)	0.0315	0.70(0.42–1.19)	0.1861
Diabetes mellitus	2.52(1.81–3.51)	< .0001	1.52(1.08–2.13)	0.0154
Liver cirrhosis	1.69(1.03–2.77)	0.0372	1.18(0.72–1.95)	0.5063
Chronic lung disease	2.01(1.26–3.20)	0.0033	1.25(0.77–2.03)	0.3591

Note: HR, hazard ratio

### Association of steroid with fracture-related and -unrelated hip arthroplasties

After adjustment for potential confounding factors (Table 3), steroid exposure was associated with excessive risks of both fracture-related (adjusted HR, 1.65; 95% CI, 1.43–1.91) and fracture-unrelated hip arthropathy (adjusted HR, 4.21; 95% CI, 3.2–5.53).

**Table 3:** Multivariate Cox proportional hazard models for hip arthroplasty with or without bone fracture.

	Fracture-related		Fracture-unrelated	
	Adjusted HR	*P*	Adjusted HR	*P*
Corticosteroid	1.65(1.43–1.91)	< .0001	4.21(3.2–5.53)	< .0001
Age (per year)	1.08(1.08–1.09)	< .0001	1.02(1.01–1.02)	< .0001
Osteoporosis	-		10.43(6.41–16.98)	< .0001
Cerebral vascular accident	0.68(0.38–1.21)	0.1880	0.67(0.21–2.15)	0.5013
Diabetes mellitus	2.16(1.53–3.05)	< .0001	0.14(0.02–1.01)	0.0514
Liver cirrhosis	1.08(0.58–2.03)	0.8104	1.71(0.76–3.87)	0.1955
Chronic lung disease	1.08(0.59–2.00)	0.7975	1.45(0.66–3.20)	0.3589

Note: HR, hazard ratio; osteoporosis was not adjusted for in the analysis for the steroid-associated risk of hip fracture, because it was involved in the mechanism underlying the association.

The risk of undergoing hip arthroplasty, whether it was related to fracture or not, was significantly higher in all steroid dosage subgroups as compared with the non-steroid cohort (Table 4). However, the risk of fracture-unrelated arthropathy rose significantly with the dose, with adjusted HR of 3.30 (95% CI, 2.44–4.46) in the low-dose, 4.54 (95% CI, 3.05–6.77) in the intermediate-dose, and 6.54 (95% CI, 4.74–9.02) in the high-dose subgroup (*P*_*trend*_<0.0001 by the Cochran–Armitage test). On the other hand, steroid was similarly associated with the surgery performed for hip fracture across all three dosage subgroups., without a dose-response relationship.

**Table 4:** Association between corticosteroid use and hip arthroplasty according to steroid dosage.

	Fracture-related		Fracture-unrelated	
	Adjusted HR	*P*	Adjusted HR	*P*
Non-users	1 (reference)		1 (reference)	
Low-dose steroid	1.52(1.29–1.79)	< .0001	3.30(2.44–4.46)	< .0001
Intermediate-dose steroid	1.68(1.30–2.17)	< .0001	4.54(3.05–6.77)	< .0001
High-dose steroid	2.00(1.63–2.46)	< .0001	6.54(4.74–9.02)	< .0001

Note: The model was adjusted for age, osteoporosis, cerebral vascular accident, diabetes mellitus, liver cirrhosis, and chronic lung disease; HR, hazard ratio; low-dose (lower quartile) indicated an average daily dose of 0.1–1.08mg, intermediate (interquartile) 1.09–13.35mg, and high dose (upper quartile) more than 13.35mg; *P*_*trend*_ by the Cochran-Armitage test was <0.0001 for arthroplasty unrelated to fracture.

## Discussion

This population-based study revealed a significant association of corticosteroid with subsequent hip arthropathy that entailed surgical intervention. Cumulatively at 12 years of follow-up, 2.96% (95% CI, 2.73–3.20%) of steroid users would require hip arthroplasty, significantly higher than 1.34% (95% CI, 1.2–1.51%) of their propensity score-matched controls (*P* < .0001). Our study further revealed that the excessive risk conferred by steroid use was more pronounced for fracture-related arthropathy, although it was also significant for hip fracture. A four-fold risk (adjusted HR, 4.21; 95% CI, 3.2–5.53) of receiving fracture-unrelated hip surgery (mainly driven by AVN) was shown in the multivariate-adjusted analysis that took age and comorbidity in to account. Moreover, the association was significant in a dose-dependent manner. Collectively, these findings contribute to bridge the knowledge gap pertaining how this extensively prescribed medication may raise the risk of serious hip diseases at a population scale. Our data not only caution against prolonged exposure to steroid, but also indicate the unmet need of effective strategies capable of preventing adverse orthopedic events in individuals who cannot spare steroid.

Osteonecrosis appears to underpin the association between steroid use and fracture-unrelated hip arthropathy[13]. This biologically plausible explanation is corroborated by the fact that AVN was the major diagnosis of hospitalization for fracture-unrelated hip surgery, although we could not radiologically or pathologically ascertain the diagnosis. Corticosteroid has been shown to inhibit angiogenesis and revascularization[13], compatible with its angiostatic property[22]. Besides, the femoral head is particularly vulnerable because of the weight-bearing stress and the reverse blood supply. Other pathogenic mechanisms linking osteonecrosis and steroid include hypertrophy of fat cells[15, 23–26], fat embolization to susceptible subchondral vessels[26, 27], and induction of cytotoxic factors[28–30]. Hypertrophy of fat cells can lead to venous obstruction, vascular occlusion, compression of small veins, and elevation of intraosseous pressure. Blood stasis, ischemia, and bone death may then ensue. Fat embolization could block the subchondral vasculature, resulting in bone ischemia and elevated intraosseous pressure.

Earlier clinical studies have been inconsistent in the association between steroid use and femoral head osteonecrosis. Similar to our findings, Vreden and colleagues found that 4 (2.4%) out of 167 patients on corticosteroid replacement therapy for adrenal insufficiency developed clinically apparent femoral head osteonecrosis[31]. By using magnetic resonance image to detect early signs of osteonecrosis, Sakamoto and colleagues reported abnormal signals in as many as 31 hips (32%) of 17 patients from a total of 48 study participants[32]. However, there were negative reports as well. For instance, Colwell and colleagues prospectively observed 71 patients who received corticosteroid for asthma or arthritis and concluded no direct relationship between corticosteroid and development of femoral head osteonecrosis after 10 years of follow-up[33]. All of these prior researches were either small in sample size or short in observation duration. More importantly, their enrollees were selected patients with a specific disease. In contrast, our study observes more than 20,000 patients for a mean duration longer than 10 years in the setting of general population.

The dose-dependent effect of steroid on the risk of fracture-unrelated hip arthropathy suggests a causal relationship. To the best of our knowledge, this is the first population-based cohort study focusing on this issue. Our study agrees with a hospital-based research conducted by Lausten and colleagues who reported that a reduction in the cumulative dose of steroid paralleled a decrease in the rate of osteonecrosis among 498 patients with renal transplantation[34]. Our results, nonetheless, should not be misinterpreted as low-dose steroid could be safe, because excessive risk was already evident in individuals who had been exposed to an average dose below 1.08 mg/day over 6 months. Clearly, it is advisable to prescribe steroid at a lowest possible dose for a shortest possible duration, if the drug is unavoidable at all.

A meta-analysis including 66 studies on BMD and 23 studies on clinical fracture concluded that oral steroid of 5mg (prednisolone equivalent) per day would lead to loss of BMD and rapidly (within 3 to 6 months) increase the risk of bone fracture[35]. Our result corroborates the association of steroid with hip fracture although the risk did not increase with dosage. Whereas the well-established osteoporotic effect of steroid would expose users to a higher long-term risk of receiving hip arthroplasty, our analyses further point out the adverse outcomes of steroid exposure can occur without an accompanying traumatic event.

The following limitations are acknowledged. First, we did not separately analysed oral or injected forms of steroid, because sequential or intermittent use of both administrative routes is common in daily practice. Second, laboratory, radiographic, and pathological data were regrettably unavailable in the NHIRD. Therefore, we resorted to admission diagnosis to define the presence of osteonecrosis. Similarly, this study could not detect subtle change of the joint or bone, such as minor fracture, but had to rely on unequivocal events to define outcomes. Nevertheless, using stringent definition and the consequence of under diagnosis would have biased the result toward null association instead of creating a spurious one, because under diagnosis was more likely to occur in those who did not seek medical attention. For instance, a diagnosis of OA would be less easily missed in patients who received steroid for relevant symptoms than in those who never used steroid. This potential misclassification should have biased the analysis toward a higher incidence of hip arthroplasty (because of undiagnosed OA) in the steroid non-users, and thus could have attenuated the association between steroid exposure and hip arthroplasty rather than have exaggerated it. Besides, this study was specifically designed to elucidate the influence of steroid, but not to comprehensively cover risk factors for hip arthropathy. Comorbid conditions were explored as covariates for the purpose of multivariate adjustment, but unmeasured confounding remained possible. The analysis could not examine potential influence by lifestyle, body weight, exercise activity, cigarette smoking, alcohol drinking, and dietary habit, since such information were unavailable in the NHIRD. As a means to overcome this limitation, we used relevant clinical diseases as surrogates. For instance, COPD is relevant for smoking and liver cirrhosis for drinking. Potential confounding by these factors were then adjusted in both the generation of propensity score and the multivariate Cox analysis.

In conclusion, corticosteroid used for 6 months or longer is associated with an increased risk of receiving hip arthroplasty in the long run. The risk results not only from hip fracture but also, more pronouncedly in fact, from arthropathy unrelated to fracture. The multivariate-adjusted association between steroid and long-term risk of fracture-unrelated hip arthropathy was significant in a dose-dependent manner. Findings from this population-based research remind healthcare professionals to minimize use of corticosteroid whenever possible. Novel strategy beyond merely strengthening the bone is needed for hip protection in patients who can’t spare steroid.

## Supporting Information

S1 Table Coding for diseases and procedures in the study. (DOCX)

S2 Table Details regarding the dosage and formula of steroid used in this study. (DOCX)
