# Supplementary material for: Corticosteroid Is Associated with Both Hip Fracture and Fracture-Unrelated Arthropathy
Source: PLoS One. 2017 Jan 26;12(1):e0169468. doi: 10.1371/journal.pone.0169468 (PMC5268437; doi:10.1371/journal.pone.0169468)
Supplement: S1 Table — (DOCX) [file pone.0169468.s001.docx]

**Supporting Information**

**S1 Table.** Coding for diseases and procedures in the study

| **Procedure** | **Corresponding ICD-9-CM codes** |
| --- | --- |
| Total hip joint replacement | 64162B |
| Hip hemiarthroplasty | 64170B |
| Hip amputation | V49.77 |
| Open reduction internal fixation for acetabulum | 64236B |
|  |  |
| **Disease** |  |
| Systemic lupus erythematous (SLE) | 710.0 |
| Rheumatoid arthritis (RA) | 714.X (X=1,2,4)  714.3Y (Y=1,2,3); |
| Avascular necrosis of femoral head | 733.42 |
| Osteoarthritis (OA) of hip joint | 715.Z5 (Z=1,2,3,9) |
| Femoral neck and femoral head fracture | 820.8; 820.9; 905.3 |
|  | 820.aa  (aa= 00;01;03;09;10;11;12;13;19) |
| acetabulum fracture | 808.0; 808.1 |
| Falling down injury (minor trauma) | E88b (b=1,2,3,4,5,6,7,8) |

Footnotes: ICD-9-CM, International Classification of Diseases, 9th Revision, Clinical Modification;
