# Supplementary material for: Corticosteroid Is Associated with Both Hip Fracture and Fracture-Unrelated Arthropathy
Source: PLoS One. 2017 Jan 26;12(1):e0169468. doi: 10.1371/journal.pone.0169468 (PMC5268437; doi:10.1371/journal.pone.0169468)
Supplement: S2 Table — (DOCX) [file pone.0169468.s002.docx]

**Supporting Information**

**S2 Table**. Details regarding the dosage and formula of steroid used in this study

| **Steroid Regimen** | | **Dosage** | **Dosing Method** |
| --- | --- | --- | --- |
| BETAMETHASONE | | 0.5 MG | Oral Tablet |
| BETAMETHASONE (SODIUM PHOSPHATE) | | 4 MG/ML | Injection |
| BETAMETHASONE (DISODIUM PHOSPHATE) | | 4 MG/ML | Injection |
| BETAMETHASONE SODIUM PHOSPHATE | | 4 MG/ML | Injection |
| BETAMETHASONE ACETATE | | 4 MG/ML | Injection |
| BETAMETHASONE ALCOHOL (DISODIUM PHOSPHATE) | | 4 MG/ML | Injection |
| CORTISONE ACETATE | | 25 MG | Oral Tablet |
| FLUDROCORTISONE ACETATE | | 0.1 MG | Oral Tablet |
| DEXAMETHASONE | | 0.5 MG | Oral Tablet |
| DEXAMETHASONE SODIUM PHOSPHATE | | 5 MG/ML | Injection |
| DEXAMETHASONE PHOSPHATE (SODIUM) | | 4 MG/ML | Injection |
| DEXAMETHASONE PHOSPHATE (21-BETA) | | 4 MG/ML | Injection |
| DEXAMETHASONE (PHOSPHATE) | | 2 MG/ML | Injection |
| DEXAMETHASONE (SODIUM PHOSPHATE) | | 5 MG/ML | Injection |
| DEXAMETHASONE (SODIUM METASULFOBENZOATE) | | 5 MG/ML | Injection |
| FRAMYCETIN (SULFATE) = SOFRAMYCIN(SULFATE) | | 5 MG/ML | Oral solution |
| GESTRINONE | | 2.5 MG | Oral Capsule |
| METHYLPREDNISOLONE (SODIUM SUCCINATE) | | 500 MG | Power Injection |
| METHYLPREDNISOLONE | | 2 MG | Oral Tablet |
| METHYLPREDNISOLONE 6-ALPHA HEMISSUCCINATE (SODIUM) | | 1000 MG | Power Injection |
| PARAMETHASONE ACETATE | | 6 MG | Oral Tablet |
| ALUMINUM BIS(ACETYLSALICYLATE)(=ALUMINUM ACETYLSALICYLA | | 150 MG | Oral Tablet |
| PREDNISOLONE | | 5 MG | Oral Tablet |
| PREDNISOLONE M-SULFOBENZOATE SODIUM | | 10 MG/ML | Injection |
| PREDNISOLONE-21-DISODIUM PHOSPHATE | | 10 MG/ML | Injection |
| PREDNISOLONE ACETATE | | 25 MG/ML | Injection |
| PREDNISOLONE PHOSPHATE SODIUM | | 1 MG/ML | Oral Solution |
| PREDNISOLONE (21-STEARYL GLYCOLATE) | 3.5 MG | | Oral Tablet |
| PREDNISOLONE SUCCINATE SODIUM | 25 MG | | Injection |
| PREDNISOLONE 21-PHOSPHATE SODIUM | 6.7 MG/ML | | Injection |
| TRIAMCINOLONE | 4 MG | | Oral Tablet |
| TRIAMCINOLONE ACETONE 21-(BENZOYLAMINE) ISOBUTYRAT | 4 MG | | Oral Tablet |
| TRIAMCINOLONE HEXACETONIDE | 20 MG/ML | | Injection |
| TRIAMCINOLONE ACETONIDE | 10 MG/ML | | Injection |
